# A participatory approach to evaluating a national training and institutional change initiative: the BUILD longitudinal evaluation

**DOI:** 10.1186/s12919-017-0082-9

**Published:** 2017-12-04

**Authors:** Pamela L. Davidson, Nicole M. G. Maccalla, Abdelmonem A. Afifi, Lourdes Guerrero, Terry T. Nakazono, Shujin Zhong, Steven P. Wallace

**Affiliations:** 1 Department of Health Policy and Management, Fielding School of Public Health, and the UCLA Clinical and Translational Science Institute, University of California, Los Angeles, CA 90095 USA; 2 Department of Education, Graduate School of Education and Information Studies, University of California, Los Angeles, CA 90095 USA; 3 Department of Biostatistics, Fielding School of Public Health and Department of Biomathematics, Geffen School of Medicine, University of California, Los Angeles, CA 90095 USA; 4 Division of General Internal Medicine/Health Services Research, David Geffen School of Medicine at the University of California, Los Angeles, CA 90095 USA; 5 Department of Community Health Sciences, Fielding School of Public Health, and UCLA Center for Health Policy Research, University of California, Los Angeles, CA 90095 USA

## Abstract

**Background and purpose:**

The National Institutes of Health (NIH) funds training programs to increase the numbers and skills of scientists who obtain NIH research grants, but few programs have been rigorously evaluated. The sizeable recent NIH investment in developing programs to increase the diversity of the NIH-funded workforce, implemented through the Diversity Program Consortium (DPC), is unusual in that it also funds a Consortium-wide evaluation plan, which spans the activities of the 10 BUilding Infrastructure Leading to Diversity (BUILD) awardees and the National Research Mentoring Network (NRMN). The purpose of this article is to describe the evaluation design and innovations of the BUILD Program on students, faculty, and institutions of the 10 primarily undergraduate BUILD sites.

**Key highlights of the project:**

Our approach to this multi-methods quasi-experimental longitudinal evaluation emphasizes stakeholder participation and collaboration. The evaluation plan specifies the major evaluation questions and key short- to long-term outcome measures (or Hallmarks of Success). The Coordination and Evaluation Center (CEC) embarked on a comprehensive evaluation strategy by developing a set of logic models that incorporate the Hallmarks of Success and other outcomes that were collaboratively identified by the DPC. Data were collected from each BUILD site through national surveys from the Higher Education Research Institute at UCLA (HERI), annual followup surveys that align with the HERI instruments, site visits and case studies, program encounter data (“tracker” data), and institutional data. The analytic approach involves comparing changes in Hallmarks (key outcomes) within institutions for biomedical students who participated versus those who did not participate in the BUILD program at each institution, as well as between institution patterns of biomedical students at the BUILD sites, and matched institutions that were not BUILD grantees. Case studies provide insights into the institutionalization of these new programs and help to explain the processes that lead to the observed outcomes.

**Implications:**

Ultimately, the results of the consortium-wide evaluation will be used to inform national policy in higher education and will provide relevant examples of institutional and educational programmatic changes required to diversify the biomedical workforce in the USA.

## Introduction: The BUILD program

In 2012, the National Institutes of Health’s (NIH) Working Group on Diversity within the Advisory Committee to the Director provided recommendations about how to develop and support individuals from diverse backgrounds across the lifespan of a research career, from undergraduate study to acquisition of tenure in an academic position or the equivalent in a non-academic setting [1]. In response, the NIH implemented a comprehensive set of actions to increase the diversity in the biomedical research workforce [2], including the funding of a Diversity Program Consortium (DPC) [3] and a rigorous evaluation of the funded programs.

**Fig. 1:**
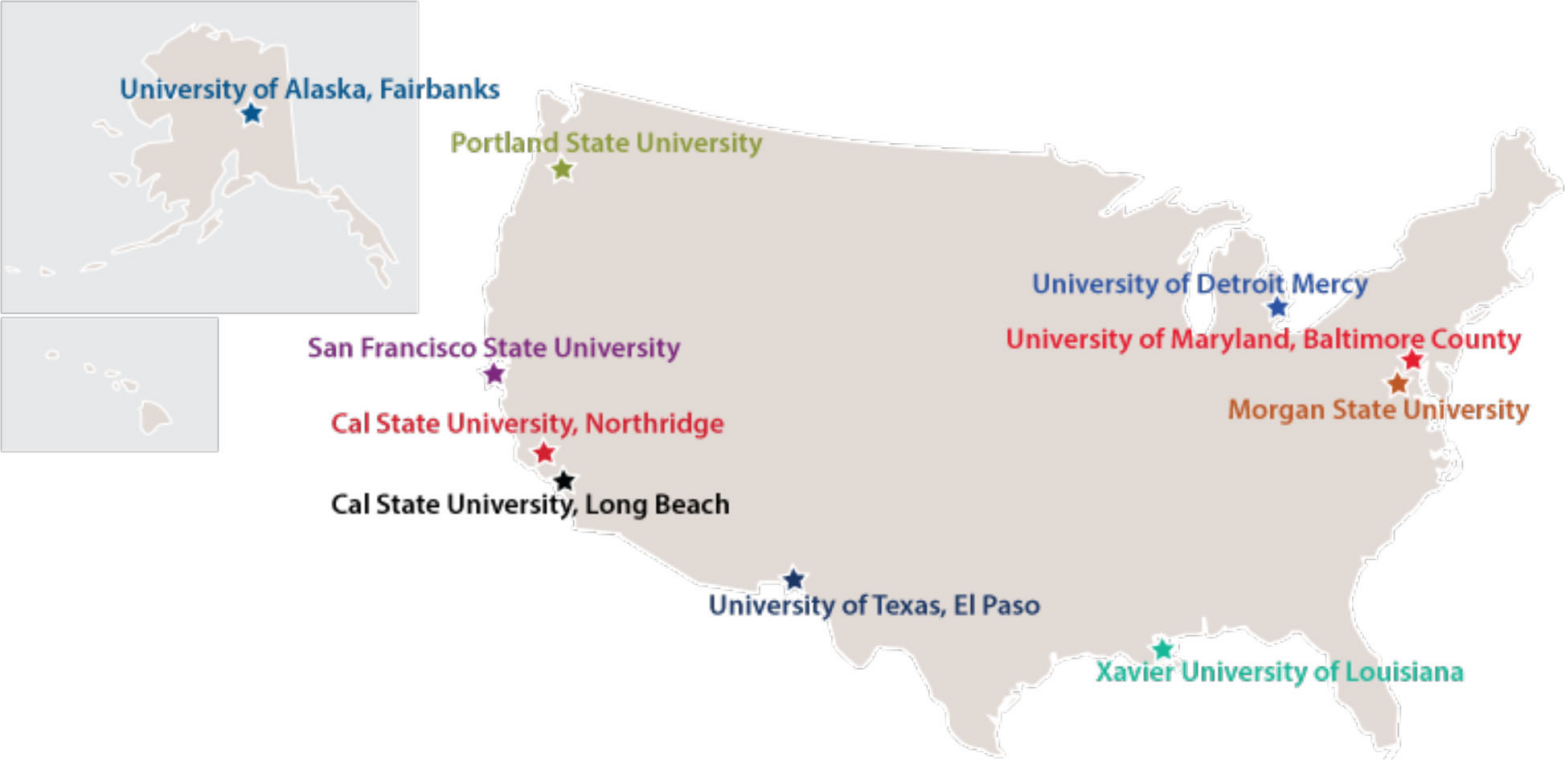
10 BUilding Infrastructure Leading to Diversity (BUILD) institutions

Figure 1 shows the geographic reach ofthe BUilding Infrastructure Leading to Diversity (BUILD) initiative, which NIH funded at 10 teaching-intensive institutions that serve high proportions of lower income and underrepresented students across the country. The BUILD institutions are implementing novel and innovative strategies to transform the undergraduate biomedical research training environment with the goal of increasing the number of students completing biomedical majors and continuing on to graduate schools with the intent of becoming biomedical researchers. BUILD sites have distinct institutional contexts, diverse student and faculty demographics, and varied partnerships with both pipeline schools (e.g. community colleges), as well as research-intensive institutions. All BUILD sites address both academic and non-academic factors that have been found to influence effective student engagement and training in biomedical fields, and that are socially and culturally responsive to the unique student populations at each institution. Additionally, sites are funded to advance the skills of biomedical faculty in research and mentoring to better support their students. BUILD programs help increase students’ science identity, sense of belonging, self efficacy, persistence and graduation in biomedical majors; promote transition to graduate studies, and set the groundwork for eventual success in biomedical research careers, particularly for those from underrepresented backgrounds. In addition, BUILD awards support institutional development to sustainably enhance the training environment through physical renovations, purchasing state of the art equipment, and curricular redesigns.

The goal of the BUILD initiative goes beyond establishing effective programs that promote biomedical workforce diversity to include research and evaluation about why different programs are effective, to experiment with novel interventions, and to enable other colleges and universities to learn from the effort. The emphasis on evaluation responds, in part, to the National Research Council’s assessment of NIH minority research training programs in 2005 that concluded that most of the information on the effectiveness of similar training programs was anecdotal, relying primarily on personal success stories [4].

BUILD programs are experimental in several ways. First, a combination of novel and existing approaches to training are being implemented at the 10 BUILD institutions. BUILD institutions have pursued a range of strategies to encourage persistence in biomedical majors. In addition to adding undergraduate research experiences, efforts include introducing new curricula, offering diversity topic workshops, providing additional academic support and counseling, connecting students with enhanced mentoring, and creating enhanced career advancement and development programs. All sites also offer a variety of faculty development activities that can make them better biomedical research teachers, including diversity training, mentor training, support for their own research, and support for course redesign. Finally, each program has an institutional change component that can involve facility renovation, new academic support programs, and new intra and inter-institutional collaborations. Since each site includes a different mix of these activities, and implements them in different ways and at different points in a student’s course of studies, there is a wide variation of program implementation that both poses a challenge for a national evaluation, but also provides rich variation to inform analyses of what works.

Lessons learned from the BUILD programs are ultimately intended to be adapted on a large scale, allowing for effective training and mentoring models to be institutionalized across various contexts for the benefit of existing and future generations of scientific talent. This comprehensive consortium wide evaluation can provide the evidence-base and report on new infrastructures, effective implementation processes and improved outcomes that may inform institutions on what works best, for whom, and in what context for training biomedical scientists from diverse groups. Successful BUILD programs may yield tangible advances and transferrable lessons in three key areas: institutional development, faculty development, and student development. The DPC BUILD initiative and requisite evaluation constitutes a landmark opportunity to study and evaluate an NIH-funded training program from planning through implementation. In addition to the Consortium wide evaluation plan, the 10 BUILD grantees are conducting site-specific evaluation plans to assess outcomes related to their distinct and novel program components.

## BUILD evaluation approach and framework

The CEC approach to the BUILD evaluation is based on years of successfully evaluating multi-site educational programs in diverse settings [5–13]. Over the decades, NSF has sponsored a number of evaluations of its programs that are designed to increase the size and diversity of the STEM workforce in large, systematic, national, cross-site evaluations [14], but NIH has been less active in this area.

A defining feature of the BUILD evaluation is its Participatory Evaluation approach, whereby program stakeholders are intricately involved in the design, implementation, and reporting/interpretation of evaluation findings, thereby increasing the appropriateness of design, the meaningfulness of the process, and the use of subsequent findings [15–18]. An early example of this approach includes the extensive collaborative work with DPC Consortium PIs around identifying Hallmarks of Success and selecting and refining key measures for inclusion in the Higher Education Research Institute (HERI) and CEC follow-up surveys that are used to address evaluation questions at the student- and faculty-level. The CEC evaluators suggested potential predictors of the outcomes that NIH had identified. DPC PI’s then added to and refined that list through a series of meetings and votes that resulted in a final set of Hallmarks (see McCreath et al. this volume). The DPC PIs subsequently discussed modifications to those hallmarks to better reflect the programs as implemented. DPC PIs and evaluators with particular expertise in specific outcomes are included as participants in the consortium-wide evaluation, and NIH program staff provide continuous feedback about the relevance and format of findings.

**Fig. 2:**
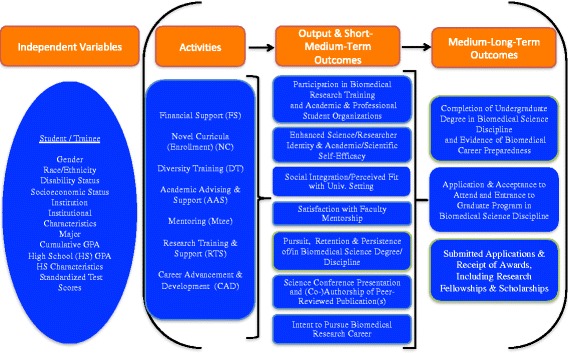
BUILD Student Logic Model

Additionally, the evaluation was grounded in Theory-Driven Evaluation whereby detailed BUILD logic models were generated based on literature reviews and the selection of Hallmarks of Success associated with successful career transitions in biomedical research [19]. Figures 2 (Student), 3 (Faculty), and 4 (Institutional) logic models were used to guide data collection and analysis, yielding important information on both implementation processes and outcomes [20–22]. The McCreath, et al., article [19], in this issue, provides more detailed information on each of the output and outcome measures (Hallmarks of Success) specified in the logic models. Extensive mapping of evaluation instruments was completed to ensure valid and reliable measures for these key outcomes of interest that were collected at each of the BUILD institutions for the Consortium-wide analyses.

**Fig. 3:**
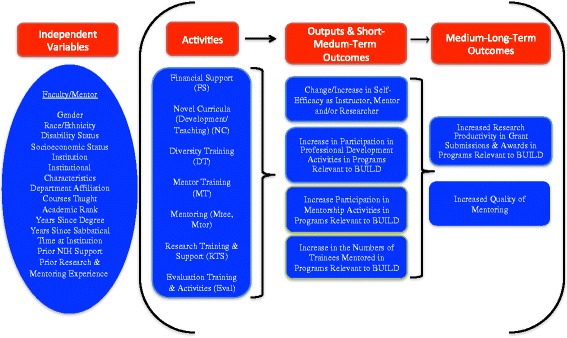
BUILD Faculty Logic Model

**Fig. 4:**
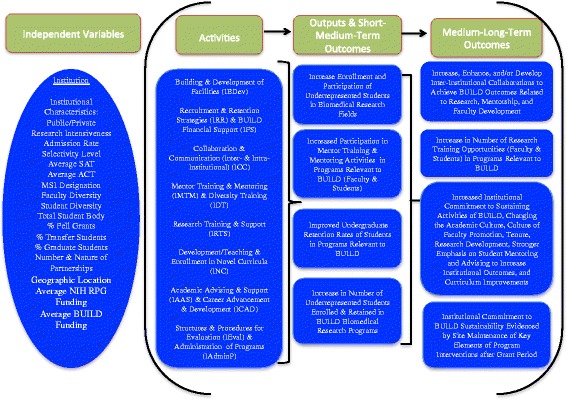
BUILD Institutional Logic Model

## BUILD evaluation design

Several key design features make the evaluation unique. These include the coordination with 10 independent site evaluations, building on a national student survey that has been in operation for 50 years [23] collaborative/longitudinal data collection, and the multi-method evaluation design essential for understanding contextual and institutional factors. In addition, this overall project provides an opportunity to examine the effects of targeting “institutions” as a method to broaden participation in biomedical research instead of strictly targeting individuals or intervention programs.

## Consortium-wide evaluation and 10 independent site evaluations

The BUILD Program specified goals at each level of the intervention. For example, student-level development goals, especially for undergraduates from underrepresented backgrounds, include increasing: (i) Science identity and self-efficacy, (ii) Social integration and perceived fit with the university setting, (iii) Intent to pursue a biomedical research career, (iv) Biomedical research experience and participation, (v) Satisfaction with faculty mentorship, (vi) Persistence/retention in biomedical science discipline, (vii) Completion of undergraduate degree in biomedical science discipline, and (viii) Application & matriculation to graduate school. McCreath, et al. [19], in this issue, provide a comprehensive list of DPC Hallmarks for the student, faculty, and institution intervention levels.

The 10 BUILD institutions each separately designed activities for their BUILD program. BUILD activities targeted at developing student capacity for biomedical research include: financial support, enrollment in redesigned curricula, diversity training, academic advising and support, mentoring, research training and support, and career advancement and development. The CEC is charged with evaluating 10 different BUILD programs, individually and collectively. In the participatory evaluation the CEC posed three broad student-level consortium wide evaluation questions that were vetted and approved by all the Consortium PIs, as follows:

1. What is the nature of BUILD activities for student development at each site and collectively across the Consortium, and how do those activities contribute to meeting the DPC student outcomes? Which activities for BUILD students have the greatest influence on DPC student outcomes?
2. To what extent are BUILD students, compared to non-BUILD students, meeting the DPC student outcomes (e.g. participation in research activities, enhanced science identity, persistence in biomedical disciplines, overall scholarly productivity, etc.)?
3. What is the student experience of BUILD activities and how does that impact program outcomes?

Similarly, consortium-wide evaluation questions were developed for the BUILD faculty and institutional levels of intervention. The primary questions posed to evaluate the BUILD faculty-level programs and activities were, as follows:

1. What is the nature of activities for BUILD faculty, and how do those activities contribute to meeting the DPC faculty outcomes? Which activities for BUILD faculty have the greatest influence on DPC faculty outcomes?
2. To what extent are BUILD faculty, compared to non-BUILD faculty, meeting the DPC faculty outcomes (e.g. participation in research activities, self-efficacy as an instructor, mentor, and researcher, overall scholarly productivity, etc.)?
3. What is the faculty experience of BUILD activities and how does that impact program outcomes?

The 10 BUILD institutions developed new activities, targeted at developing faculty capacity for biomedical research, including: financial support, development and/or teaching of novel curricula, diversity training, mentor training and mentoring, and opportunities for research experience, training and support.

The primary questions posed to evaluate the BUILD institution-level programs and activities were, as follows:

1. Do the number and/or diversity of students graduating in biomedical sciences in BUILD institutions increase over time?
2. How have BUILD and partner institutions developed the capacity for biomedical science research training and mentoring and in what ways is this sustainable?
3. How have BUILD institutions embraced organizational changes that promote institutional commitment to diversity?

Build institutions designed activities aimed at strengthening institutional capacity for biomedical research, including: new strategies for recruitment and retention of diverse students and faculty, improved strategies for collaboration and communication with internal and external partners, and building and development of facilities, as well as structures and procedures to implement activities geared specifically towards students and faculty.

Individual sites were primarily responsible for conducting their own formative and process evaluations, which provided them with the most tailored and timely information about how their programs were deployed early on and the implications for early modifications. Most sites have innovative components that are unique to one or a few sites, such as student and faculty trainings on stereotype threat, or interinstitutionally aligned coursework and seamless transfer policies. The evaluation of the effectiveness of those particular intervention components are the primary responsibility of individual BUILD Programs. The national evaluation focuses on the Consortium-wide Hallmarks which are the core outcome measures that all sites have oriented some programming to address. Since there are a number of Hallmarks (e.g. science identity, biomedical major persistence), that may be improved by those unique program components, the evaluation design enables the merging of site-specfic and national evaluation data to improve our understanding of the implementation processes involved.

As a mechanism for aligning the 10 BUILD site level evaluations and the consortium level evaluation, we convened annual consortium wide meetings and periodic meetings of all the Consortium evaluators. The evaluators convened two-day in person meetings to review progress towards both site specific and DPC evaluation goals, share strategies for evaluation data collection and analysis, and to foster cross-site collaborations in the evaluation of outcomes that were intervention targets at a subset of sites, e.g., reducing stereotype threat. Finally, monthly meetings were held regarding Consortium-wide data collection with each BUILD site and monthly webinars were convened to showcase best practices in implementation of interventions and evaluation strategies.

Local methods for evaluating the student experiences at each campus vary according to the characteristics of the BUILD students, faculty, and institutions, and the actual program activities deployed. For example, evaluating student experiences at the smaller, rural feeder campuses that are part of the Biomedical Learning and Student Training (BLaST) Program at University of Alaska-Fairbanks (UAF) is different from those at the two larger campuses in Fairbanks and Juneau. UAF is using participatory action research projects to engage both communities and students in biomedical research in rural/remote Alaska to better reflect subsistence community values [24, 25]. Thus, the more indepth site level evaluations are complimentary and address questions that are context specific, focus on local differences in BUILD student/faculty diversity, and implementation processes.

## Building on a National Survey: Longitudinal assessment and tracking

A strength of the Consortium-wide evaluation is the use of existing national surveys administered by the Higher Education Research Institute (HERI) at the UCLA Graduate School of Education & Information Studies and the aligned supplemental CEC annual follow-up surveys.

The national evaluation draws on three HERI surveys: The Freshman Survey (baseline survey), the College Senior Survey, and a Faculty Survey [26]. The CEC conducts Annual Follow-up Surveys to those who answer The Freshman Survey and the Faculty Surveys. The Consortium-level evaluation and each of the BUILD sites added up to 20 survey items to reflect the Consortium Hallmarks and the BUILD intervention and outcome variables to each HERI survey. HERI student data is available at a large number of institutions for the freshman year and at graduation, and for faculty every three years. To follow interim changes, the CEC also created annual follow-up surveys that focus on the relevant Hallmarks that were administered in the years that the HERI surveys are not answered by continuing students and in the interm years for faculty. The baseline on students and faculty (for BUILD and non-BUILD participants) and CEC followup surveys provide longitudinal data for local evaluation needs and consortium-wide evaluation reports.

To be able to match changes in Hallmark outcomes with BUILD program activities, the CEC also developed a “tracker” to identify student and faculty participation in the program activities. Sites upload rosters of relevant activities (classes, research trainings, mentor/mentee pairings, etc.), which are then merged with individual student and faculty IDs to create a comprehensive administrative record of level of exposure to BUILD interventions. This avoids reporting errors that can occur if participants self-report program activities but do not realize what is actually part of the BUILD program (e.g. redesigned classes), or when they may have forgotten an encounter since it occurred months before the annual survey (e.g. a workshop); it also significantly reduces respondent burden for those involved in many BUILD activities. This allows the national evaluation to create measures of intensity of BUILD exposure, and also to better isolate different types of BUILD exposure (e.g. research experience vs. financial support) for analysis. Tracker data is also available to each BUILD site for their local evaluations. The campuses are experimenting with a tracker app that allows easy tracking of participation in BUILD activities, and geofencing capabilities for student participation in program events.

Additionally, the tracker reflects an extensive document review of programmatic materials (presentations, program websites, promotional materials, site-level evaluation plans, communications, etc.), yielding a rich repository of the range and novel features of activities offered on each campus. The various interventions across BUILD programs were mapped into a single summary document (“at-a-glance”), giving an overview of unique program activity features within and across BUILD programs. This permitted a comprehensive overview of campus-based activities and their evolution, as well as a way to target evaluation to similar components and determine areas suitable for coordinated evaluation efforts. Campuses upload and refine their intervention activities in order to keep track of student participation and later determine impact.

## Collaborative/longitudinal data collection

This collaborative longitudinal evaluation requires rigorous and replicable collection and use of data. The 10 BUILD sites collaborate with the CEC to collect the consortium wide longitudinal data, both quantitative and qualitative, but as indicated above, also conduct a local site level evaluation that is complementary to the consortium wide study. Figures 2-4 present the Consortium logic models including the output and outcome constructs (Hallmarks of Success), that all the sites collect via HERI and CEC Follow-up surveys; these comparable measures enable cross-site analysis and comparisons among student and faculty cohorts.

The multi-levels of sampling and longitudinal evaluation enable comparisons: (1) within sites of BUILD students and faculty to non-BUILD students and faculty; (2) across 10 BUILD institutions to examine the varying effects of program activities on improving the Hallmarks of Success; and (3) of BUILD institutions with a matched sample of non-BUILD institutions to determine the effectiveness of the BUILD program overall. Additionally, as indicated above, linking the CEC tracker data to individual student and faculty data within BUILD sites allows us to compare level of exposure that is required (no exposure, small exposure, intensive exposure) to achieve the Hallmarks of Success.

Among the major challenges in conducting the evaluation is the non-response from students who are at the core of BUILD – students from groups underrepresented in biomedical fields [27]. We used the participatory evaluation approach to address the critical need to generate rigorous, robust, comparable data for the national evaluation, which led to on-going discussions with BUILD sites about how to maximize response rates of both students and faculty. In communications with BUILD sites, three major priorities emerged to improve response rates, including: (1) Incentives: we had general agreement among sites that cash/gift card incentives make a difference; however, the challenge was determining how best to use resources, especially in minimizing attrition in the longitudinal data collection; (2) Timing: the CEC is collaborating with each BUILD site to schedule a survey administration window to avoid proximity/overlap with other university surveys or major institutional requirements (e.g. final exams); (3) Communication to potential participants: Our discussions with the BUILD site evaluators centered on “Selling participation” by using tactics, such as publicizing surveys more on campus, using social media, highlighting the importance of getting the institutional leaders and the broader student community involved to encourage students to participate, highlighting the importance of their participation in this national effort and its implications for other students like them, increasing the number of follow-up reminders to as many as four contacts, and closely tracking responses from the BUILD students and faculty. Later examples included extensive site-level coordination of data collection procedures (securing access to individuals for sampling, customizing email invitations for participants, and garnering high-level administrative support for participating in evaluation activities, etc.).

The team structure worked well to align the site level data collection with the Consortium-wide evaluation and facilitated an effective bi-directional communication between the BUILD sites and the CEC. Since the BUILD sites were also conducting site level evaluations in addition to participating in the Consortium-level evaluation, having sufficient personnel and resources for completing the scope of work at both these levels was challenging. The CEC addressed the challenge by working collaboratively with BUILD evaluators to streamline data collection. This reduced redundancies in data collection, giving preference to standardized Consortium measures that were valid, reliable, and comparable across sites, and addressed the Hallmarks of Success.

The most important aspect of the longitudinal data collection and analysis was to address the gaps in the evaluation literature on interventions. NIH wanted to improve the evidence base by better understanding what drives workforce changes 10–15 years in the future in terms of diversifying the nations biomedical research thinkforce. By following the students over 5–10 years it will be able to identify which of the Hallmarks (increased science identity, better mentoring, stronger peer communities, etc.), are correlated with increasing persistence in biomedical undergraduate majors, graduation, and enrollment in graduate school. Following these students on their career trajectory through their doctorate and post-doctoral fellowships will inform us if the interventions in the undergraduate years alone persist for students from underrepresented groups, and the extent to which additional support through graduate school and their early career acts as a booster, or supplement, to the undergraduate experience. This longitudinal design is crucial for being able to identify the key changes needed to support the evolution of the NIH scientific “thinkforce” to better represent the demographics of the US population.

## Multi-method evaluation design

We have implemented a multi-method evaluation design that emphasizes multiple case studies, cross-site analysis, a robust quasi-experimental design using multilevel statistical analysis to compare BUILD and non BUILD-exposed students and faculty, as well as BUILD and non-BUILD sites, and trend analysis of key indicators, e.g., Hallmarks of Success. Table 1 summarizes the BUILD multi-methods longitudinal evaluation design in terms of data sources, evaluation questions, sample, data collection/timing, and data analysis. All data sources and timelines for data collection are aligned to answer the key evaluation questions at the student, faculty, and institutional levels.

**Table 1:** Summary of BUILD Multi-methods Longitudinal Evaluation Design

	Data Sources	Evaluation Question(s)^a^	Sample	Data Collection/ Timing (grant years)	Data Analysis
Student	HERI: The Freshman Survey (TFS)	S:2	- Representative sample (race/ethnicity) of 500 Students per year, per institution for 10 BUILDs (*n* = 5000 per year, per survey), non-proportional based on major (80% Biomedical major, 20% non-Biomedical major), mix of BUILD/non-BUILD exposure (all student surveys) (ICS only for rising Juniors and Seniors). - Comparable non-BUILD sample from non-BUILD institutions for TFS and CSS (*n* = TBD)	Fall: Y2-Y5	- Multilevel analysis - Assess degree of exposure effect of BUILD intervention
	HERI: College Senior Survey (CSS)			Spring: Y3-Y5	
	CEC BUILD Interim College Survey (ICS)			Fall: Y3	
	CEC BUILD Student Annual Follow-up Survey (S-AFS), rising Sophomores, Juniors, Seniors			Spring: Y3-Y5	
Faculty	HERI Faculty Survey (FAC)	F:2	- 50 Faculty per institution for 10 BUILDs (*n* = 500 total), all from Biomedical disciplines, mix of BUILD/non-BUILD exposure - Comparable non-BUILD sample from non-BUILD institutions for FAC (*n* = TBD)	Fall: Y3	- Multilevel analysis - Assess degree of exposure effect of BUILD intervention
	CEC BUILD Faculty Annual Follow-up Survey (F-AFS)			Spring: Y3-Y5	
	Mentee-Mentor Assessment Survey		- Student/Mentee assessment of mentor (*n* = 1–2 mentees per BUILD faculty, per year)	Spring: Y3-Y5	
Cross Cutting (Student, Faculty, Institutional)	Integrated Postsecondary Education System (IPEDS) Data	S:2; F:2; I:1	- 10 BUILDs and comparable sample from matched non-BUILD institutions	Pre-Implementation and Y1-Y5	- Descriptive analysis
	Offices of Institutional Research (IR) Data	S:2; F:2; I:1	- 10 BUILDs	TBD with Sites Y1-Y5	- Multilevel analysis
	Program Implementation Data (CEC Tracker)	S:1; S:2; F:1; F:2		Ongoing Y1-Y5	- Descriptive analysis
	Site Visit – Interviews	S:3; F:3; I:2; I:3	- 10 BUILDs (including Pipeline & Research Partners)	Y3-Y4	- 4-phase coding process - Steps to ensure credibility, trustworthiness, & inter-rater reliability
	Site Visit – Observations	S:3; F:3; I:2; I:3			
	Document Analysis	S:1; F:1; I:2; I:3	- 10 BUILDs (Websites, Presentations, Reports, etc.)	Ongoing Y1-Y5	

^a^Evaluation Questions at the Student (S), Faculty (F), and Institutional (I) Levels, as outlined in the body of the manuscript

A multi-method evaluation design was essential for understanding contextual and institutional factors that shape biomedical training and outcomes. All 10 BUILD institutions participate in site visits during the grant period. Site visits focus on describing the activities BUILD sites implement to promote and support underrepresented groups biomedical research training at each site. Using the *site level* BUILD program logic model as a guiding framework, qualitative interviews were conducted to provide a narrative description of the relationships among each BUILD site’s inputs, activities and outputs and some, but not all, short-term program outcomes. Qualitative data collection was focused on the approaches for forming institutional partnerships, as well as to capture the participant experience within the BUILD program (e.g., PI, faculty, and student). Site visits were an occasion for sites to showcase the defining features of their programs, their model for organizational transformation and sustainability, as well as to discuss any challenges related to program implementation and evaluation. Additionally, a series of case studies were conducted to capture institutional transformation and sustainability of BUILD program features and structural changes.

## Summary of data analytic strategy

There are two sets of comparisons that will be used to determine if the BUILD programs are effective, one internal to BUILD institutions and one comparing BUILD to non-BUILD institutions. The first set of analyses compares those involved with the BUILD program at each site with similar biomedical students and faculty who are not involved with the BUILD programs at the same institution. In tracking changes over time for both groups, we will be able to determine if (after adjusting for selection biases between the two populations) students and faculty in the BUILD programs perform better than those who are not in the program (otherwise known as a difference in difference approach). Because we will have detailed information about which BUILD activities each participant was exposed to, we can also determine if there is a minimum extent or type of participation needed for improved outcomes. The variation between sites also will allow us to examine variation in program design, such as the timing of student research experiences, the creation of student learning communities, or the extent of faculty research support provided. Since the ultimate goal of the program is to increase the diversity of the biomedical workforce, we will also look at the interaction of underrepresented group membership with the intervention to determine the relative effectiveness for underrepresented students and faculty.

Since one of the goals of BUILD is institutional-level change, it is possible that even students and faculty who were not direct participants in BUILD activities may benefit from campus-wide changes that the BUILD programs stimulate. To better isolate institutional-level changes we have also selected a national matched sample of non-BUILD institutions that also collected HERI student surveys at the same time as each of the 10 BUILD grantees. Matching of BUILD/non-BUILD institutions was based on 2015 data for the following six indicators: Public vs. private college or university; Percent undergraduates receiving a Pell grant; Percent of applications deemed admissible to the institution; Average SAT scores; Percent under-represented students (Black, Latino/Hispanic, Native-American); and institution size measured by total number of undergraduates. Therefore, each BUILD site has a comparable institution whose students and faculty can be tracked in terms of key Hallmarks over time.

Secondary data from HERI surveys at those non-BUILD institutions will be used for comparison with outcomes at BUILD institutions for students at baseline (The Freshman Survey) and near graduation (College Senior Survey), and for faculty surveyed every three-years (Faculty Survey). In this analysis we will examine key outcomes at the individual level to determine if being at a BUILD versus non-BUILD institution has significantly more impact on biomedical career outcomes.

Generalized mixed (fixed and random effects) linear models (multilevel models) are used to test the main hypothesis that the BUILD interventions result in better outcomes. In analyses, we adjust for individual (students and faculty) and contextual (institutional) characteristics. We also account for the clustering effect, namely that data from students and faculty in the same institution may be more similar to each other than those in other institutions.

Testing institutional hypotheses will employ both quantitative and qualitative methods.

In order to answer the first BUILD institutional evaluation question, institutional data from 2013 (one to two years pre-implementation, depending on planning grant awards) to 2019 (end of funding), for all 10 BUILD grantee institutions will be used. Aggregate data for all students in biomedical disciplines will be examined for changes in entry, persistence, and graduation rates across the life of the grant. Increases in total numbers and/or diversity (e.g. underrepresented groups based on race/ethnicity, disability, and/or socio-economic status (SES) for students in biomedical majors/disciplines will be determined. Individual student data will be analyzed using a generalized logistic mixed effects model that calculates the relative probability that students at BUILD institutions will choose and graduate in a biomedical major. Covariates will include whether the student is from an underrepresented group, predispositions, and experiences of each student at the institution.

A qualitative approach was implemented to answer most of the BUILD institutional evaluation questions and some evaluation questions for students and faculty. The qualitative data were collected during site visits (semi-structured interviews, observations, and relevant program documents) in the form of institutional case studies. BUILD sites were debriefed regarding key observations relevant to improving program implementation. Analysis of qualitative data moves through the phases of organizing, coding, sorting, synthesizing, and theorizing [28]. Strategies to increase credibility and trustworthiness of the data and ensure high inter-rater reliability were implemented [29, 30]. Case studies were designed to provide richly contextualized data that can be compared along several dimensions to determine common and unique aspects of institutional processes and impact [31] to answer instituitional research questions regarding commitment to diversity, implementation, and sustainability.

Table 2 demonstrates some of the complexity inherently involved in the BUILD national longitudinal evaluation, based on the wide variation in institutional contexts. Not only are programs geographically dispersed, but so too are the program activity features and contexts in which programs are being implemented. There is a mix of public, private, HBCU, and minority serving institutions with major differences in selectivity levels and their history of receiving funding from NIH. There is great range in student body size, graduation rates, and focus on serving undergraduate, transfer, and graduate students. This rich diversity in programmatic and institutional contexts will continue to guide evaluation design, implementation, and analysis of initiative and program impact.

**Table 2:** BUILD Institutional Contextual Data

BUILD Prime Grantee Institution (State)	Public/ Private/ HBCU	Average Total NIH Funding 2011-2013^A^	Admission Rate^B^	Average SAT^C^	Percent Pell Grants^D^	6-yr. Graduation Rates	Total Student Body	Percent URM^E^	Number of Incoming Freshman	Percent Transfer Students
California State University, Long Beach (CA)	Public	$4.3	35%	1055	50%	57%	36,809	42%	4335	12%
California State University, Northridge (CA)	Public	$4.7	53%	915	52%	48%	40,131	48%	5526	16%
San Francisco State University (CA)	Public	$7.9	66%	990	43%	47%	29,465	29%	3754	12%
Portland State University (OR)	Public	$5.2	69%	1030	41%	42%	27,696	15%	1703	15%
University of Alaska Fairbanks (AK)	Public	$8.8	73%*	545*	29%	33%	8620	20%	942	6%
University of Texas at El Paso (TX)	Public	$11.3	100%	460*	58%	38%	23,079	85%	3256	10%
Xavier University of Louisiana (LA)	HBCU	$5.0	66%	1005	53%	47%	2976	79%	579	5%
University of Detroit Mercy (MI)	Private	$0.0	69%	1121	29%	57%	4945	14%	469	7%
University of Maryland, Baltimore County (MD)	Public	$9.0	60%	1210	28%	61%	13,979	22%	1629	11%
Morgan State University (MD)	HBCU	$1.6	65%	880	62%	29%	7698	87%	1078	7%

Note. Information regarding institutional characteristics is pulled from the Institute of Education Sciences (IES) National Center for Education Statistics (NCES) - Final release data, 2013-2016. Items with a * are from https://www.princetonreview.com/. See: https://nces.ed.gov/ipeds/datacenter/ BUILD Program names are listed under each of the BUILD Chapters in this issue

^A^Context of NIH funding at BUILD institutions, reported in Millions, prior to BUILD awards. Retrieved from NIH Project Reporter, 2011-2015. See: https://projectreporter.nih.gov/reporter.cfm

^B^Selectivity level based on admission rates, is defined as follows: Less Competitive: more than 65%, Moderately Competitive: between 51%-65%, Competitive: between 34%-50%, Most Competitive: between 16%-33%, Elite or Highly Competitive: Less than 15%

^C^Average SAT score is calculated based on the average of the 25th and 75th percentile for Critical Reading and Math. Average scores are only reported for institutions with data available for 25% or more of their undergraduate students

^D^Percentage of Pell Grant for all undergraduate students or first time enrolled (UAF only) undergraduate students

^E^Percentage of Underrepresented Minorities (URM) is the sum of the percent of undergraduate students from the following ethnicities: American Indian or Alaskan Native; Black or African American; Hispanic/Latino; and Native Hawaiian or Other Pacific Islander. These rates are likely underestimated given the exclusion of percent two or more races and unknown ethnicity

## Challenges and limitations

We have addressed five major challenges and limitations in conducting a multi-campus program evaluation. First, expertise varies widely at the BUILD teaching intensive institutions to collect systematic data for the evaluation. This has implications for valid and reliable measurement. We addressed this by forming the 10 CEC-BUILD Teams that supported the needed expertise across all the sites. Second, the critical importance of providing adequate incentives to stimulate response rates both among students and faculty longitudinally in both intervention and comparison groups; prior evaluations have been hampered by low response rates that yield biased evaluation findings. We optimized response rates through a 3-pronged approach that used incentives, timing of survey administration, and more effective communication strategies.

Third, selection bias is always inherent in the quasi-experimental design due to baseline differences in institutional level and individual level faculty and student samples. We addressed this by using statistical controls for baseline differences in intervention and comparison groups, to the extent possible. Fourth, collecting longitudinal data for assessing career success in biomedical research many years into the future from the undergraduate program through the key transition points of tenured faculty or comparable scientific position [1] is threatened by the difficulties of long-term longitudinal tracking and the associated threat to internal validity known as attrition. In the short-term we have taken measures to optimize response rates; in the longer term, controlling for attrition in longitudinal studies is often problematic. We will continue to use incentives, persuasive communications, and develop a national alumni of BUILD scholars who will meet annually to stay connected and in communication. And, fifth, identifying the key programmatic activities in the context of implementing multiple program components and activities simultaneously at each site poses the threat to external validity known as the multiple treatments interference. Our CEC tracker data will be used to tease out the influence of specific BUILD activities implemented at each site to address this threat to external validity.

## Implications/conclusions

This article describes the conceptual and methodological foundation of the BUILD evaluation design to determine the national impact of this institutionally-targeted initiative. The results of the Consortium-wide evaluation will be used to inform national policy and institutional and educational programmatic changes required to diversify the biomedical workforce in the USA.

The early evaluation of the 10 BUILD programs describe the characteristics of students, faculty, and institutions attracted to the programs, and documents the extent to which the NIH DPC investment is working to change early predictors of success among underrepresented students in remaining and succeeding in biomedical majors in college. Since the BUILD educational interventions and innovations did not start until year two of the five-year grants, and successful student graduation is typically measured over a four to six-year period, the first round of evaluation will focus largely on early predictors such as science identity development and satisfaction with mentoring. It will also position the longer-term evaluation that will allow us to follow students through graduation and into graduate school,or the labor force, so that we can both track successful outcomes (e.g. matriculation into a biomedical graduate program) and determine if the literature on predictors of success apply to the students in these teaching-intensive institutions with high numbers of students who are underrepresented in the biomedical workforce. The results will provide rich information on how to best fine-tune future programs to address the most important predictors of biomedical success in these types of institutions. Because we have information from 10 different programs, the results will also provide insights into the types of programs that have the greatest impact on the recruitment, retention, and matriculation to graduate programs in the biomedical sciences of underrepresented students.

The evaluation is unique in that it not only focuses on student outcomes but also focuses on the program effects among faculty and institutions. Impacting the faculty and institutions are essential for sustaining the program effects beyond the fixed years of BUILD’s NIH funding. The BUILD evaluation data will show the types of programs that have the greatest effect on improving faculty mentoring of biomedical students, as well as how they work to change the culture, infrastructure, and/or standard operating processes of the institutions in ways that better support URG success in biomedical majors in sustainable ways.

Ultimately, the success of the evaluation is dependent on the level of cooperation and coordination across various stakeholder groups that are geographically dispersed and the effective use of communication technology to convene virtual meetings and webinars to form and sustain high performing multi-campus working groups at all levels of the consortium. Educating a biomedical workforce that represents the diversity of the U.S in the 21^st^ century requires the broad implementation of evidence-based approaches. The Consortium-wide evaluation is a key component for building the evidence base to identify best in class career development interventions to address gaps in biomedical workforce diversity.
